# Ca**^+2^**/Calmodulin-Dependent Protein Kinase Mediates Glucose Toxicity-Induced Cardiomyocyte Contractile Dysfunction

**DOI:** 10.1155/2012/829758

**Published:** 2012-06-18

**Authors:** Rong-Huai Zhang, Haitao Guo, Machender R. Kandadi, Xiao-Ming Wang, Jun Ren

**Affiliations:** ^1^Department of Geriatrics, Xijing Hospital, The Fourth Military Medical University, Xi'an 710032, China; ^2^Department of Physiology, The Fourth Military Medical University, Xi'an 710032, China; ^3^Division of Pharmaceutical Sciences, University of Wyoming, College of Health Sciences, Laramie, WY 82071, USA

## Abstract

(1) Hyperglycemia leads to cytotoxicity in the heart. Although several theories are postulated for glucose toxicity-induced cardiomyocyte dysfunction, the precise mechanism still remains unclear. (2) This study was designed to evaluate the impact of elevated extracellular Ca^2+^ on glucose toxicity-induced cardiac contractile and intracellular Ca^2+^ anomalies as well as the mechanism(s) involved with a focus on Ca^2+^/calmodulin (CaM)-dependent kinase. Isolated adult rat cardiomyocytes were maintained in normal (NG, 5.5 mM) or high glucose (HG, 25.5 mM) media for 6-12 hours. Contractile indices were measured including peak shortening (PS), maximal velocity of shortening/relengthening (±dL/dt), time-to-PS (TPS), and time-to-90% relengthening (TR_90_). (3) Cardiomyocytes maintained with HG displayed abnormal mechanical function including reduced PS, ±dL/dt, and prolonged TPS, TR_90_ and intracellular Ca^2+^ clearance. Expression of intracellular Ca^2+^ regulatory proteins including SERCA2a, phospholamban and Na^+^-Ca^2+^ exchanger were unaffected whereas SERCA activity was inhibited by HG. Interestingly, the HG-induced mechanical anomalies were abolished by elevated extracellular Ca^2+^ (from 1.0 to 2.7 mM). Interestingly, the high extracellular Ca^2+^-induced beneficial effect against HG was abolished by the CaM kinase inhibitor KN93. (4) These data suggest that elevated extracellular Ca^2+^ protects against glucose toxicity-induced cardiomyocyte contractile defects through a mechanism associated with CaM kinase.

## 1. Introduction

 Hyperglycemia in individuals with diabetes mellitus usually compromises myocardial contractile function and energy metabolism independent of macro- and microvascular coronary anomalies [1–4]. Hyperglycemia-associated pathological changes in the heart are characterized by myocardial damage, cardiac hypertrophy, overt fibrosis, structural and functional changes of myocardium, and cardiac autonomic neuropathy [4]. A number of theories have been postulated for hyperglycemia-induced myocardial dysfunction including direct glucose toxicity, impaired glucose metabolism, disrupted energy metabolism, oxidative stress, and interrupted intracellular Ca^2+^ homeostasis [4–8]. Despite the fact that these factors may contribute to cardiac contractile anomalies and tissue damage in hyperglycemic condition, the ultimate cause responsible for the hyperglycemia- and diabetes-triggered myopathic change remains elusive. Recent evidence has suggested a role of impaired energy metabolism and energy reserve in hyperglycemia-associated cardiac contractile impairment [9, 10]. Along the same line, reports from our laboratory as well as others have revealed that the cell energy fuel AMP-dependent protein kinase (AMPK) and the essential energy substrate pyruvate protect against cardiomyocyte contractile dysfunction under hyperglycemic or metabolic derangement conditions [11–13]. However, the precise nature behind AMPK and pyruvate-improved cardiac contractile function under hyperglycemic or diabetic condition remains unclear.

Ca^2+^/calmodulin-dependent protein kinase II (CaMKII), a serine-threonine protein kinase implicated in a variety of cardiovascular regulation, was found with high activity in brains under diabetes, possibly contributes to neuronal cell death [14, 15]. Interestingly, CaMKII also promotes cell survival in response to various stresses [16, 17], thus making CaMKII an important point of intersection for different pathways involved in diseases. To this end, this study was designed to examine the impact of elevated extracellular Ca^2+^ levels mimicking a higher cardiac energy supply on cardiomyocyte contractile function and intracellular Ca^2+^ handling in cardiomyocytes maintained in normal glucose (NG) or high glucose (HG) environment. Levels and activity of the intracellular Ca^2+^ regulatory proteins including sarco(endo)plasmic reticulum Ca^2+^-ATPase (SERCA), phospholamban, and Na^+^/Ca^2+^ exchanger were examined.

## 2. Methods

### 2.1. Isolation and Culture of Rat Cardiomyocytes

The experimental procedures used in this study were approved by the Animal Use and Care Committee at the University of Wyoming (Laramie, WY, USA). In brief, adult male Sprague-Dawley rats (200–250 g) were anesthetized using ketamine/xylazine (5 : 3, 1.32 mg/kg i.p.). Hearts were rapidly removed and perfused (at 37°C) with the Krebs-Henseleit bicarbonate (KHB) buffer (mM: NaCl 118, KCl 4.7, MgSO_4_ 1.2, KH_2_PO_4_ 1.2, NaHCO_3_ 25, N-[2-hydro-ethyl]-piperazine-N′-[2-ethanesulfonic acid] (HEPES) 10, glucose 11.1; pH 7.4). The heart was perfused for 20 min with KHB containing 176 U/mL collagenase II (Worthington Biochemical Corp., Freehold, NJ, USA) and 0.5 mg/mL hyaluronidase. After perfusion, left ventricles were removed and minced. The cells were further digested with 0.02 mg/mL trypsin before being filtered through a nylon mesh (300 *μ*m). Extracellular Ca^2+^ was added incrementally back to 1.25 mM. Isolated cardiomyocytes were cultured on glass coverslips precoated with laminin (10 *μ*g/mL) and maintained for 6 or 12 hours in a defined medium consisting of Medium 199 with Earle's salts containing 25 mM HEPES and NaHCO_3_ supplemented with albumin (2 mg/mL), L-carnitine (2 mM), creatine (5 mM), taurine (5 mM), insulin (0.1 *μ*M), penicillin (100 U/mL), streptomycin (100 *μ*g/mL), and gentamicin (100 *μ*g/mL). This medium also contained either normal glucose (NG: 5.5 mM) or high glucose (HG: 25.5 mM) concentrations. The high glucose concentration is comparable to serum glucose levels in severely diabetic rats [18]. Subsets of NG and HG media were supplemented with the glycation inhibitor aminoguanidine (1 mM) [19] or the translation inhibitor cycloheximide (60 *μ*m) [20]. DMSO was used as a solvent with a final concentration less than 0.5%. These inhibitors were added at the time when myocytes were placed in medium, and cells were incubated for 12 hours at 37°C under 100% humidity and 5% CO_2_ [21].

### 2.2. Cell Shortening/Relengthening

Mechanical properties of cardiomyocytes were assessed using a video-based edge-detection system (IonOptix Corporation, Milton, MA, USA) as described previously [21]. In brief, cardiomyocytes were superfused at 25°C with a buffer containing (in mM) 131 NaCl, 4 KCl, 1 CaCl_2_, 1 MgCl_2_, 10 glucose, and 10 HEPES, at pH 7.4. The cells were field stimulated at 0.5 Hz. Cardiomyocytes were displayed on a computer monitor using a Pulnix variable speed camera, which rapidly scans image areas every 8.3 msec such that the amplitude of shortening/relengthening is recorded with good fidelity. A cohort of cardiomyocytes was recorded in the above-mentioned recording buffer with the exception of a higher extracellular Ca^2+^ level at 2.7 mM in the absence or presence of the CaM kinase inhibitor 2-[*N*-(2-hydroxyethyl)]-*N*-(4-methoxybenzenesulfonyl)amino-*N*-(4-chloro-cinnamyl)-*N*-methylbenzylamine (KN93, 10 *μ*M) [20].

### 2.3. Intracellular Ca^2+^ Transients

Isolated cardiomyocytes were loaded with fura-2/AM (0.5 *μ*M) for 10 min. Fluorescence intensity was recorded using a dual-excitation fluorescence photomultiplier system (IonOptix). Cardiomyocytes were placed onto an Olympus IX-70 inverted microscope and imaged through a Fluor 40x oil objective. Cells were exposed to light emitted by a 75 W lamp and passed through either a 360 or a 380 nm filter while being stimulated to contract at 0.5 Hz. Fluorescence emissions were detected between 480 and 520 nm, and qualitative change in fura-2 fluorescence intensity was inferred from the fura-2 fluorescence intensity ratio at the 2 wavelengths (360/380). Fluorescence decay time was calculated as an indicator of intracellular Ca^2+^ clearance [22].

### 2.4. Western Blotting

Protein samples for western blotting were prepared as described [22]. Briefly, cardiomyocytes were homogenized and sonicated in a lysis buffer containing 20 mM Tris (pH 7.4), 150 mM NaCl, 1 mM ethylenediaminetetraacetic acid (EDTA), 1 mM ethyleneglycoltetraacetic acid (EGTA), 1% Triton, 0.1% SDS, and 1% protease inhibitor cocktail. Equal amounts (50 *μ*g) of proteins were separated on 10%–12% SDS-polyacrylamide gels in a minigel apparatus (Mini-PROTEAN II, Bio-Rad Laboratories Inc., Hercules, CA, USA) and were then transferred electrophoretically to nitrocellulose membranes. The membranes were blocked with 5% milk in Tris-buffered saline before overnight incubation at 4°C with anti-SERCA2a (1 : 1,000), anti-Na^+^-Ca^2+^ exchanger (1 : 1,000), anti-phospholamban (1 : 1,000), and anti-GAPDH (1 : 1,000) antibodies. Membranes were then incubated for 1 hr with horseradish peroxidase- (HRP-) conjugated secondary antibody (1 : 5,000). All other antibodies were obtained from the Cell Signaling Technology (Beverly, MA, USA). After immunoblotting, the film was scanned, and the intensity of immunoblot bands was detected with a Bio-Rad Calibrated Densitometer. GAPDH was used as the loading control.

### 2.5. SERCA Activity Measured by ^45^Ca^2+^ Uptake

Cardiomyocytes were sonicated and solubilized in a Tris-sucrose homogenization buffer consisting of 30 mM Tris-HCl, 8% sucrose, 1 mM PMSF and 2 mM dithiothreitol, pH 7.1. To determine SERCA-dependent Ca^2+^ uptake, samples were treated with and without the SERCA inhibitor thapsigargin (10 *μ*M) for 15 min. The difference between the two readings was deemed the thapsigargin-sensitive uptake through SERCA. Uptake was initiated by the addition of an aliquot of supernatant to a solution consisting of (in mM) 100 KCl, 5 NaN_3_, 6 MgCl_2_, 0.15 EGTA, 0.12 CaCl_2_, 30 Tris-HCl pH 7.0, 10 oxalate, 2 ATP, and 1 *μ*Ci ^45^CaCl_2_ at 37°C. Aliquots of samples were injected onto glass filters on a suction manifold and washed 3 times. Filters were then removed from the manifold, placed in scintillation fluid and counted. SERCA activity was expressed as cpm/mg protein [23].

### 2.6. Statistical Analysis

For each experimental series, data are presented as Mean ± SEM. Statistical significance (*P* < 0.05) for each variable was estimated by analysis of variance (ANOVA).

## 3. Results

### 3.1. Effect of Short-Term Culture on Adult Rat Cardiomyocyte Mechanical Properties

**Figure 1:**
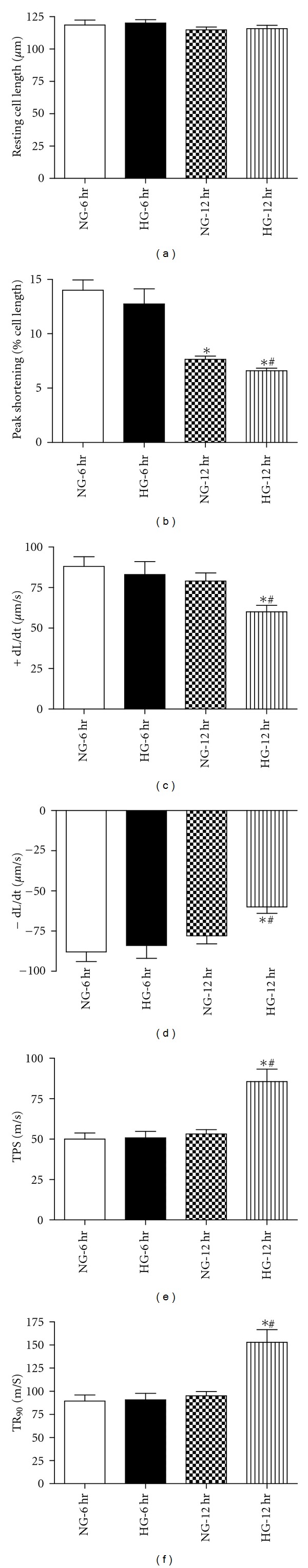
Mechanical property of adult rat cardiomyocytes cultured for 6 and 12 hours in a serum-free medium with normal glucose (NG: 5.5 mM) or high glucose (HG: 25.5 mM). (a) Resting cell length, (b) peak shortening (PS) amplitude normalized to cell length, (c) maximal velocity of shortening (+dL/dt), (d) maximal velocity of relengthening (−dL/dt), (e) time to PS (TPS), and (f) time to 90% relengthening (TR_90_). Mean ± SEM, *n* = 24 and 41-42 cells per group at 6 and 12 hours, respectively, *P < 0.05 versus NG-6 Hr group, ^#^ *P* < 0.05 versus NG-12 Hr group.

Our data shown in Figure 1 revealed that 6 hours of incubation of high extracellular glucose did not affect any of the cell mechanics evaluated. Interestingly, prolonged culturing in normal glucose medium significantly diminished peak shortening (PS) amplitude without affecting the resting cell length, maximal velocity of shortening/relengthening (±dL/dt), time to PS (TPS), and time to 90% relengthening (TR_90_). Following 12 hours of incubation, high glucose medium significantly decreased PS and ±dL/dt, as well as prolonged TPS and TR_90_.

### 3.2. Impact of Glycation or Translation Inhibition on High Glucose-Induced Cardiomyocyte Mechanical Anomalies

**Figure 2:**
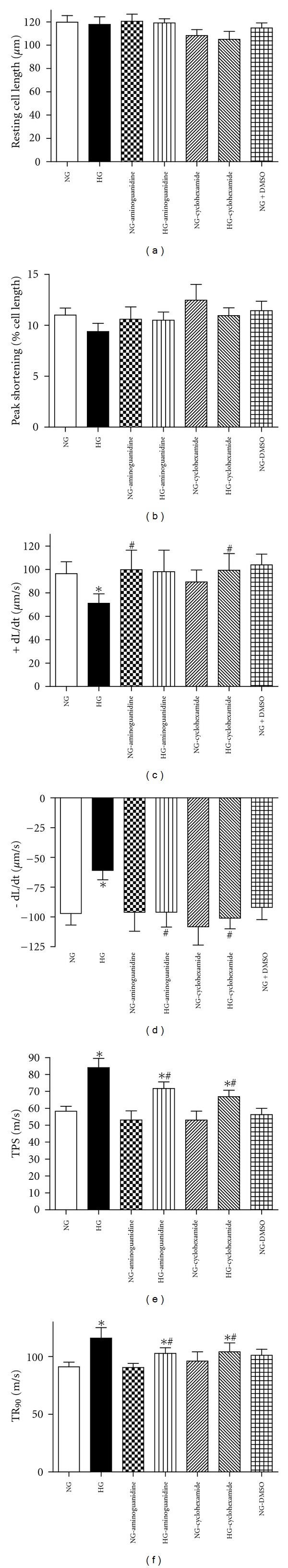
Mechanical property of adult rat cardiomyocytes cultured for 12 hours in a serum-free medium with normal glucose (NG: 5.5 mM) or high glucose (HG: 25.5 mM) in the absence or presence of the glycation inhibitor aminoguanidine (1 mM) or the translation inhibitor cycloheximide (60 *μ*m). DMSO was used as a solvent with a final concentration less than 0.5%. (a) Resting cell length, (b) peak shortening (PS) amplitude normalized to cell length, (c) maximal velocity of shortening (+dL/dt), (d) maximal velocity of relengthening (−dL/dt); (e) time to PS (TPS), and (f) time to 90% relengthening (TR_90_). Mean ± SEM, *n* = 22–29 cells per group, *P < 0.05 versus NG group, ^#^ *P* < 0.05 versus HG group.

 To examine the potential mechanism(s) behind the high glucose-induced cardiomyocyte contractile abnormalities, the glycation inhibitor aminoguanidine (1 mM), or the translation inhibitor cycloheximide was coincubated with adult rat cardiomyocytes for 12 hours maintained in either normal or high glucose medium. Our data depicted that both aminoguanidine and cycloheximide significantly attenuated or mitigated the high glucose-induced cardiomyocyte mechanical anomalies (without affecting resting cell length). Neither inhibitor affected cardiomyocyte contractile properties by itself (Figure 2). These data depicted a potential role of glycation and protein translation in glucose toxicity-induced cardiomyocyte contractile defects.

### 3.3. Impact of Elevated Extracellular Ca^2+^ Concentration on High Glucose-Induced Cardiomyocyte Mechanical and Intracellular Ca^2+^ Handling Derangements

**Figure 3:**
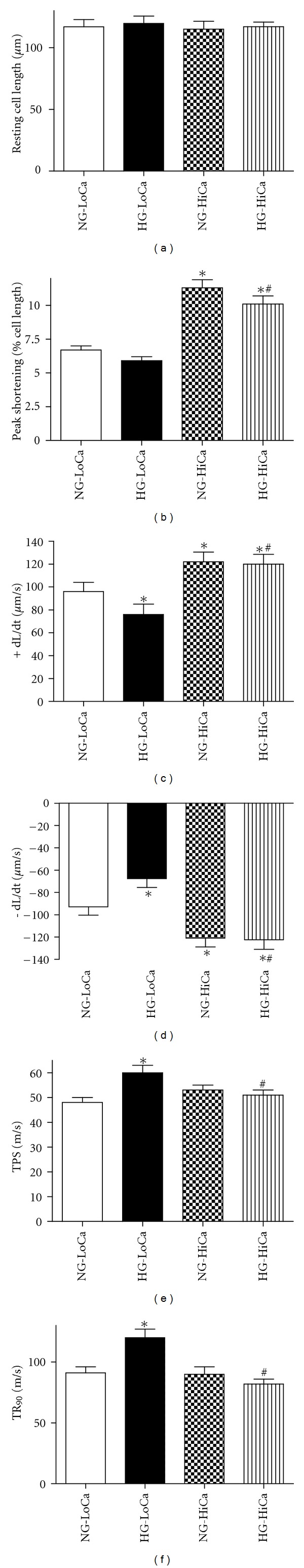
Mechanical property of adult rat cardiomyocytes maintained for 12 hours in a serum-free medium with normal glucose (NG: 5.5 mM) or high glucose (HG: 25.5 mM) in the absence or presence of high extracellular Ca^2+^ (Hi-Ca, 2.7 mM) in the contractile buffer. An extracellular Ca^2+^ concentration of 1.0 mM was used as the normal low Ca^2+^ (LoCa) environment. (a) Resting cell length (b) peak shortening (PS) amplitude normalized to cell length; (c) Maximal velocity of shortening (+dL/dt); (d) Maximal velocity of relengthening (−dL/dt), (e) time to PS (TPS), and (f) time to 90% relengthening (TR_90_). Mean ± SEM, *n* = 38–42 cells per group, *P < 0.05 versus NG-LoCa group, ^#^ *P* < 0.05 versus HG-LoCa group.

**Figure 4:**
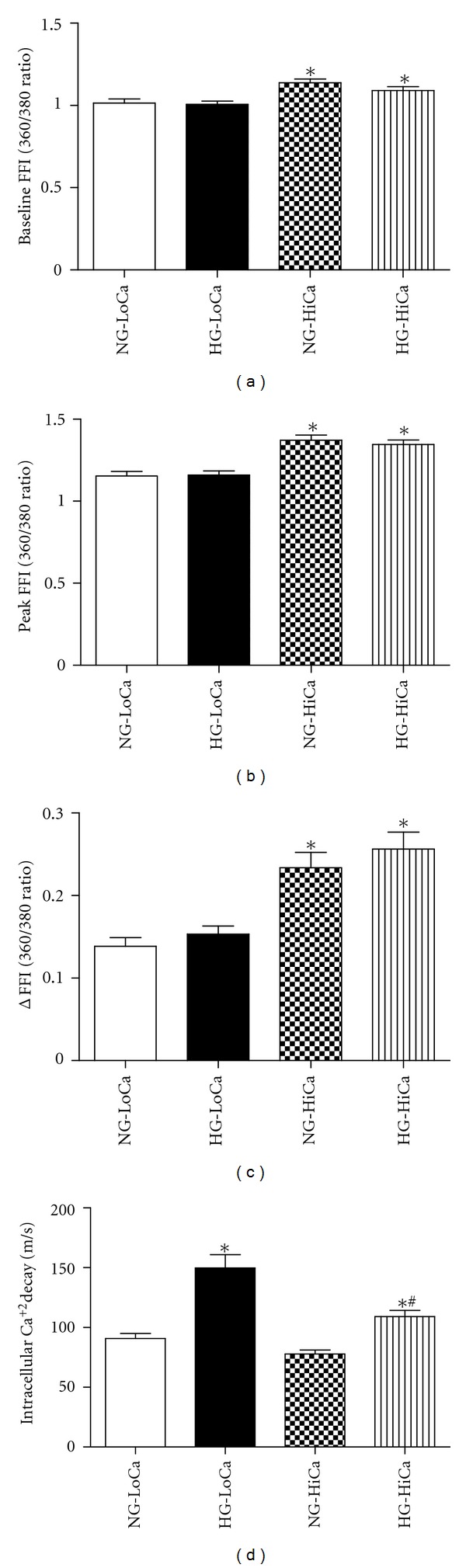
Intracellular Ca^2+^ handling property of adult rat cardiomyocytes cultured for 12 hours in a serum-free medium with normal glucose (NG: 5.5 mM) or high glucose (HG: 25.5 mM) in the absence or presence of high extracellular Ca^2+^ (Hi-Ca, 2.7 mM) in the contractile buffer. An extracellular Ca^2+^ concentration of 1.0 mM was used as the low Ca^2+^ (LoCa) environment. (a) Resting intracellular Ca^2+^ levels shown as baseline fura-2 fluorescent intensity (FFI), (b) peak FFI, (c) electrically stimulated rise of FFI (ΔFFI), and (d) intracellular Ca^2+^ decay rate. Mean ± SEM, *n* = 29–42 cells per group, *P < 0.05 versus NG-LoCa group, ^#^ *P* < 0.05 versus HG-LoCa group.

The above-mentioned data were collected under an extracellular Ca^2+^ concentration of 1 mM. To evaluate if facilitated intracellular Ca^2+^ dynamics with elevated extracellular Ca^2+^ levels on high glucose-induced response in cardiomyocyte mechanics, Ca^2+^ concentration was raised from 1 mM to 2.7 mM in the recording contractile buffer. Data shown in Figure 3 revealed that elevated extracellular Ca^2+^ significantly improved cardiomyocyte contractile capacity in cells maintained in both normal and high glucose environments. PS and ±dL/dt were overtly elevated in response to elevated extracellular Ca^2+^ level associated with unchanged resting cell length, TPS, and TR_90_. Interestingly, high glucose-induced abnormalities in cardiomyocyte contractile abnormalities seen at the extracellular Ca^2+^ level of 1 mM were abolished when extracellular Ca^2+^ levels were raised to 2.7 mM. To evaluate the potential mechanism of action underlying high extracellular Ca^2+^-induced beneficial effect against glucose toxicity-induced cardiomyocyte mechanical responses, intracellular Ca^2+^ handling was evaluated using the fura-2 fluorescence dye. Our data indicated that high extracellular Ca^2+^ promoted intracellular Ca^2+^ handling in both normal and high glucose environments [elevated resting, peak and change of fura-2 fluorescence intensity (FFI)] associated with unchanged intracellular Ca^2+^ decay. Similar to the mechanical response, elevated extracellular Ca^2+^ levels effectively attenuated or nullified glucose toxicity-induced derangement of intracellular Ca^2+^ handling (delayed intracellular Ca^2+^ clearance with unchanged baseline, peak and increase in FFI) (Figure 4).

### 3.4. Impact of Elevated Extracellular Ca^2+^ on High Glucose-Induced Changes in Intracellular Ca^2+^ Regulatory Proteins in Cardiomyocytes

**Figure 5:**
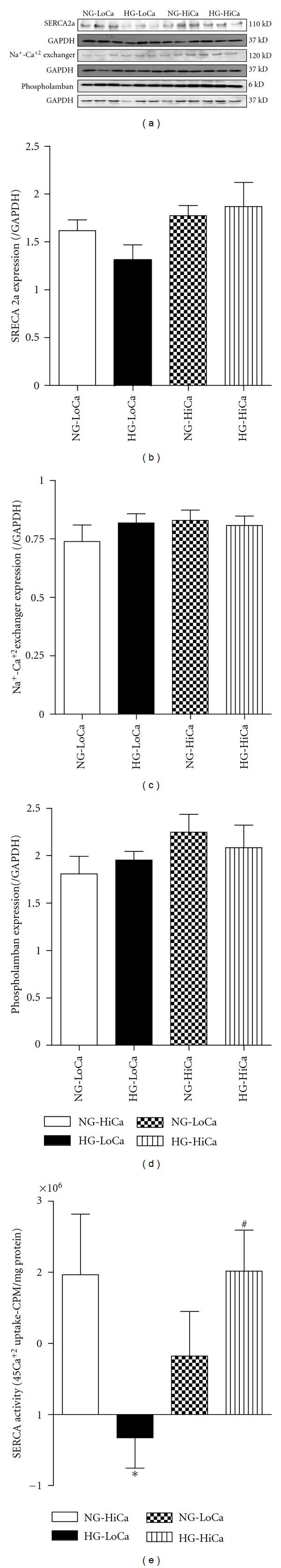
Expression of intracellular Ca^2+^ regulatory proteins in rat cardiomyocytes cultured for 12 hours in a serum-free medium with normal glucose (NG: 5.5 mM) or high glucose (HG: 25.5 mM) in the absence or presence of high extracellular Ca^2+^ (Hi-Ca, 2.7 mM) in the recording contractile buffer. An extracellular Ca^2+^ concentration of 1.0 mM was used as the normal low Ca^2+^ (LoCa) environment. (a) Representative gel blots depicting levels of SERCA2a, Na^+^-Ca^2+^ exchanger, phospholamban, and GAPDH (loading control), (b) SERCA2a expression, (c) Na^+^-Ca^2+^ exchanger expression, (d) phospholamban expression, and (e) SERCA activity measured using ^45^Ca^2+^ uptake. Mean ± SEM, *n* = 6-7 isolations per group, *P < 0.05 versus NG-LoCa group, ^#^ *P* < 0.05 versus HG-LoCa group.

To further evaluate elevated extracellular Ca^2+^ and high glucose-induced changes in intracellular Ca^2+^ handling, expression of intracellular Ca^2+^ regulatory protein was evaluated. Our results revealed that neither high extracellular Ca^2+^ nor high glucose, or both, produced any notable changes in the levels of SERCA2a, Na^+^-Ca^2+^ exchanger and phospholamban. However, measurement of SERCA activity revealed that high extracellular glucose significantly dampened the thapsigargin-sensitive ^45^Ca^2+^ uptake under low extracellular Ca^2+^ environment, the effect of which was nullified by elevated extracellular Ca^2+^. Last but not least, increase in extracellular Ca^2+^ concentration itself did not affect expression or activity of intracellular Ca^2+^ regulatory proteins (Figure 5).

### 3.5. Effect of CaM Kinase Inhibition on Elevated Extracellular Ca^2+^-Induced Protection against Glucose Toxicity

**Figure 6:**
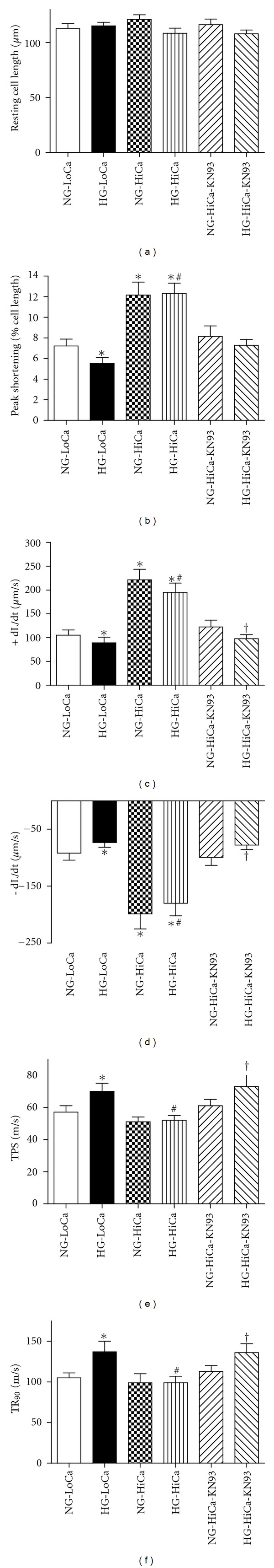
Mechanical property of rat cardiomyocytes cultured for 12 hours in a serum-free medium with normal glucose (NG: 5.5 mM) or high glucose (HG: 25.5 mM) in the absence or presence of high or low extracellular Ca^2+^ (Hi-Ca = 2.7 mM; LoCa = 1.0 mM) in the recording contractile buffer. A cohort of HG-cultured cardiomyocytes was recorded in high Ca^2+^ environment in the presence of the CaM kinase inhibitor KN93 (10 *μ*M). (a) Resting cell length, (b) peak shortening (PS) amplitude normalized to cell length, (c) maximal velocity of shortening (+dL/dt), (d) maximal velocity of relengthening (−dL/dt), (e) time to PS (TPS), and (f) time to 90% relengthening (TR_90_). Mean ± SEM, *n* = 10 cells per group, *P < 0.05 versus NG-LoCa group, ^#^ *P* < 0.05 versus HG-LoCa group, ^†^ *P* < 0.05 versus NG-HCa-KN93 group.

 Elevated extracellular Ca^2+^ levels are known to activate CaM kinase. To evaluate if CaM kinase plays a role in the elevated extracellular Ca^2+^ concentration-induced beneficial effect against glucose toxicity, cardiomyocytes maintained in normal or high glucose medium were recorded in low or high extracellular Ca^2+^ environments in the absence or presence of the CaM kinase inhibitor KN93 (10 *μ*M). Our data revealed that KN93 effectively abolished high extracellular Ca^2+^-offered protection against high glucose-induced cardiomyocyte contractile anomalies (decrease in PS and ±dL/dt as well as prolonged TPS and TR_90_). In addition, KN93 also abolished high extracellular Ca^2+^-induced cardiomyocyte contractile responses (increased PS and ±dL/dt along with unchanged TPS and TR_90_) under low extracellular Ca^2+^ environment (Figure 6).

## 4. Discussion

Result from our study revealed that elevated extracellular Ca^2+^ level mitigated the glucose toxicity-induced cardiomyocyte mechanical and intracellular Ca^2+^ anomalies. Glucose toxicity or elevated extracellular glucose level has been shown to compromise cardiomyocyte contractile function and intracellular Ca^2+^ homeostasis following short-term exposure [24, 25], suggesting a role of hyperglycemia in cardiac contractile anomalies. Interestingly, our finding revealed that the high extracellular Ca^2+^-induced benefit effect against glucose toxicity was abolished by the CaM kinase inhibitor KN93, suggesting a role of CaM kinase in elevated extracellular Ca^2+^-induced beneficial effect against glucose toxicity.

Hyperglycemia has been shown to be one of the most devastating predisposing factors for the onset and development of diabetic cardiomyopathy [2–4, 24]. Reduced cardiac contractility, depressed maximal velocity of contraction/relaxation, and prolonged contraction/relaxation are considered landmarks of hyperglycemia or diabetes-induced myopathic alterations [2, 4, 7, 8, 24]. Findings from this study confirmed our earlier observations that cardiomyocytes maintained in high glucose medium for 12 hours (but not 6 hours) compromised cardiomyocyte mechanical properties manifested as depressed peak shortening and maximal velocity of shortening/relengthening, along with prolonged duration of contraction and relaxation, in a manner similar to *in vivo* diabetes [6, 24, 25]. The subtle difference in contractile capacity in NG-maintained cardiomyocytes (Figures 1 and 6) may be largely due to interculture and/or individual murine variations. Earlier studies from our laboratory have established that normal cardiomyocytes may display diabetes-like phenotype of cardiac mechanical dysfunctions simulating *in vivo* diabetes after only 12–24 hours of culture in a serum-free high glucose medium [18, 24, 25]. The use of isolated cardiomyocyte model allows the precise control of the extracellular milieu in which cardiomyocytes reside, thus maximally eliminating the possible interference from fibroblasts, endothelial metabolism, and diffusion barriers [25–27]. Intriguingly, the glycation inhibitor aminoguanidine and the translation inhibitor cycloheximide attenuated glucose toxicity-induced cardiomyocyte contractile defects without eliciting any effect by themselves. These findings suggest possible involvement of glycation and protein translation in glucose toxicity-induced cardiac contractile anomalies. Earlier findings from our laboratory as well as others have depicted a pivotal role of glycation in hyperglycemia and diabetes-induced cardiac dysfunctions [5, 6, 28, 29]. Furthermore, our data revealed that glucose toxicity-induced cardiomyocyte anomalies may be abolished with elevated extracellular Ca^2+^ levels (from the physiological level of 1.0 mM to 2.7 mM), the effect of which may be abolished by inhibition of CaM kinase using KN93. Several mechanisms may be speculated for elevated extracellular Ca^2+^-induced protection against glucose toxicity. Elevated extracellular Ca^2+^ levels are capable of facilitating cytosolic Ca^2+^ cycling and dynamics thus promoting Ca^2+^ influx and resequestration, regardless of the extracellular glucose environment. This is supported by the increased rise of intracellular Ca^2+^ in response to electrical stimuli, facilitated intracellular Ca^2+^ decay, improved SERCA activity (although not expression), and shortened TR_90_ under high extracellular Ca^2+^ environment. Our observation of depressed cardiomyocyte contractile capacity and maximal velocity of contraction/relaxation along with prolonged duration of contraction and relaxation in high glucose-treated cardiomyocytes is supported by compromised intracellular Ca^2+^ handling under high glucose environment, consistent with the findings from *in vivo *diabetic condition [8, 26]. Another scenario may be related to changes in cardiac energy dynamics through CaM kinase signaling cascade. Ca^2+^ ions promote cell survival through a CaM kinase-dependent activation of Akt [30], in line with the finding from our current study. If energy states were the sole determinant of cardiac contractile function, then normal glucose-cultured cardiomyocytes should display improved cardiac contractile performance, consistent with our experimental finding. One of the main mechanisms that extracellular Ca^2+^ improves cardiac performance is through increased phosphorylation state. It may be speculated that Ca^2+^ ions promote contractile inotropism first by bolstering cardiac energy state, which occurs rapidly within minutes. The CaM kinase cascade is essential to many physiological processes, the defect of which can lead to a variety of disease states. Calmodulin acts as a primary receptor for Ca^2+^ in all eukaryotic cells whereas Ca^2+^/CaM kinase functions as an allosteric activator of a host of enzymatic proteins [15]. The area CaM-dependent kinase cascade is just on the horizon. Further investigation on the cellular machineries in particular cardiac excitation-transcription coupling regulated by this intracellular Ca^2+^-initiated signaling cascade should hold considerable promise for the future of disease-related research [31].

In summary, the findings from the present study revealed improved cardiac contractile and intracellular Ca^2+^ homeostasis against glucose toxicity. Considering the unique role of CaM kinase in regulation of cardiac energy metabolism and survival, understanding the mechanism of action of CaM kinase in myocardial contractile regulation under pathological conditions such as hyperglycemia should be essential in the elucidation of CaM kinase in diabetic complications.
